# Synthesis of Trimeric Organozinc Compounds and their Subsequent Reaction with Oxygen

**DOI:** 10.1002/open.201600040

**Published:** 2016-07-15

**Authors:** Joe A. Manzi, Caroline E. Knapp, Ivan P. Parkin, Claire J. Carmalt

**Affiliations:** ^1^ Department of ChemistryUniversity College London20 Gordon StreetLondonWC1H 0AJUK

**Keywords:** precursors, structure elucidation, thin films, trimeric compounds, zinc

## Abstract

A conventional solution‐based route to a cyclic trimeric organozinc compound [{Zn(Et)(*β*‐diketonate)}_3_] (*β*‐diketonate=OC(OMe)CHC(Me)O, **1**) is described, with **1** structurally characterized for the first time. The ligand selection of bidentate *β*‐diketonates is shown to be key to isolating a cyclic trimer. Additional reaction of *β*‐diketonates with diethyl zinc were spectroscopically characterized as compounds of the type [{Zn(Et)(*β*‐diketonate)}_*n*_] (*β*‐diketonate=OC(Me)CHC(Me)O, **2**, OC(O*t*Bu)CHC(Me)O, **3**). Further studies have shown that selective oxidation of these species produces cubanes of the general formula [{Zn(OC(R)CHC(Me)O)_2_Zn(Et)OEt}_2_] (R=OMe, **4**; Me, **5**; O*t*Bu, **6**), allowing a high oxygen content whilst remaining structurally suitable for use as precursors. The successful deposition of thin films of zinc oxide through aerosol‐assisted chemical vapor deposition (AACVD), using a novel precursor, is described and fully characterized.

Studies involving zinc alkoxides were first reported by Frankland in 1849^1^ and the complex chemistry of alkyl zinc reactivity with oxygen has been revealed in the literature over the last century.^2^ Organozinc compounds find use as catalysts, for example, in ring‐opening polymerizations,^3^, ^4^ as epoxidizing agents for enones,^5^ and as precursors for nanoparticle synthesis^6^ and zinc oxide functional thin films (including doping in main‐group materials).^7^

The widely accepted belief that the oxidation of zinc alkyls is so fast, selectivity is not possible, was first dispelled by the structural characterization of a novel zinc alkylperoxide.^8^ Since then, the field has undergone a renaissance, owing to re‐examination of the oxidation of R_2_Zn compounds through the contributions of Lewiński et al. and a handful of other groups.^9^, ^10^, ^11^ A study reported by Lewiński et al. in 2003 unequivocally proved that selective oxidation of organozinc compounds is possible, with the synthesis of [EtOOZn‐ (*azol*)]_2_[EtZn(*azol*)]_2_, (*azol*=deprotonated 1‐aziridineethanol, a centrosymmetric cluster including octahedral‐ and tetrahedral‐coordinated zinc environments) bridged by peroxide groups. Interestingly, this work was the first to ‘suggest the presence of a trimer as the predominate species in solution’ of the precursor aggregate, namely, [EtZn(*azol*)]_*n*_.^12^

It is known that the structure of organozinc compounds can be enormously varied, including multinuclear rings^11^ or cubanes,^13^ and DFT studies have even considered cyclic and roof‐like structures (Figures 1 a and 1 b).^14^

**Figure 1:**
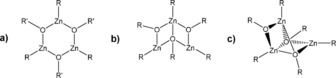
Central Zn_3_O_3_ motifs: a) cyclic, b) roof‐like, and c) cluster.

A recent study using a range of flexible donor‐functionalized amino alcohols reported a variety of dimeric and tetrameric clusters.^10^, ^15^ It was proposed through an extensive NMR study that [EtZn(bdmap)]_3_ (bdmap:1,3‐*bis*(dimethylamino)‐2‐(dimethylaminomethyl)‐*iso*‐propoxide), whilst not crystallographically characterized, has a cyclic, not a roof‐like, trimeric structure (Figure 1); although, mass spectral data suggested a more complex scenario.

In 2010, the first crystallographically characterized organozinc ‘dimeric aggregate’ with a Zn_3_O_3_ core cluster (Figure 1 c)) was published.^16^ This was synthesized through a solid‐state transformation of [{*t*BuZn(*μ*‐O*t*Bu)(thf)}_2_] under mild conditions to give [{*t*BuZnO*t*Bu}_3_]. This cluster was reported to have two distinct types of organozinc centers: two four‐coordinate zinc centers linked by two *μ*_3_‐ and one *μ*_2_‐oxygen atoms, and one three‐coordinate zinc center binding to two *μ*_2_‐oxygen atoms. It was found to be a metastable product, as solution‐based attempts to form the compound resulted solely in the tetramer. Later in 2012, it was shown that by using a more rigid quinoline ligand, thus moving from a ZnCO_3_ coordination environment to ZnCNO_2_, a trimer could be isolated and was structurally characterized as [*t*BuZn(q)]_3_ (q=8‐hydroxyquinoline).^17^

Herein, we report the first instance of a conventional solution‐based synthetic route to a structurally characterized cyclic trimeric organozinc compound with the coordination environment ZnCO_3_ (Figure 1 a), [{Zn(Et)(OC(OMe)CHC(Me)O)}_3_] (**1**), in which the use of bidentate *β*‐diketonate ligands facilitated crystallization. Similar reactions of diethyl zinc with *β*‐diketonates yielded compounds of the type [{Zn(Et)(*β*‐diketonate)}_*n*_], which were spectroscopically characterized. In addition, selective reaction of these compounds with oxygen yields distorted cubanes of the general formula [{Zn(*β*‐diketonate)_2_Zn(Et)OEt}_2_]. These compounds exhibit a similar central motif to that reported previously.^12^, ^17^, ^18^

The trimeric *β*‐diketonate [{Zn(Et)(OC(OMe)CHC(Me)O)}_3_] (**1**) was isolated and structurally characterized; furthermore, compounds of stoichiometry [{Zn(Et)(OC(R)CHC(Me)O)}_*n*_], where R=Me (**2**) and O*t*Bu (**3**) (Scheme 1), were confirmed spectroscopically. Compounds **1**–**3** were isolated from the equimolar reaction of Et_2_Zn with the respective carbonyl in high yield (>90 %) and characterized by using spectroscopic techniques. Although **2** and **3** formed crystalline solids, none were of suitable quality for crystallographic analysis.

**Scheme 1:**
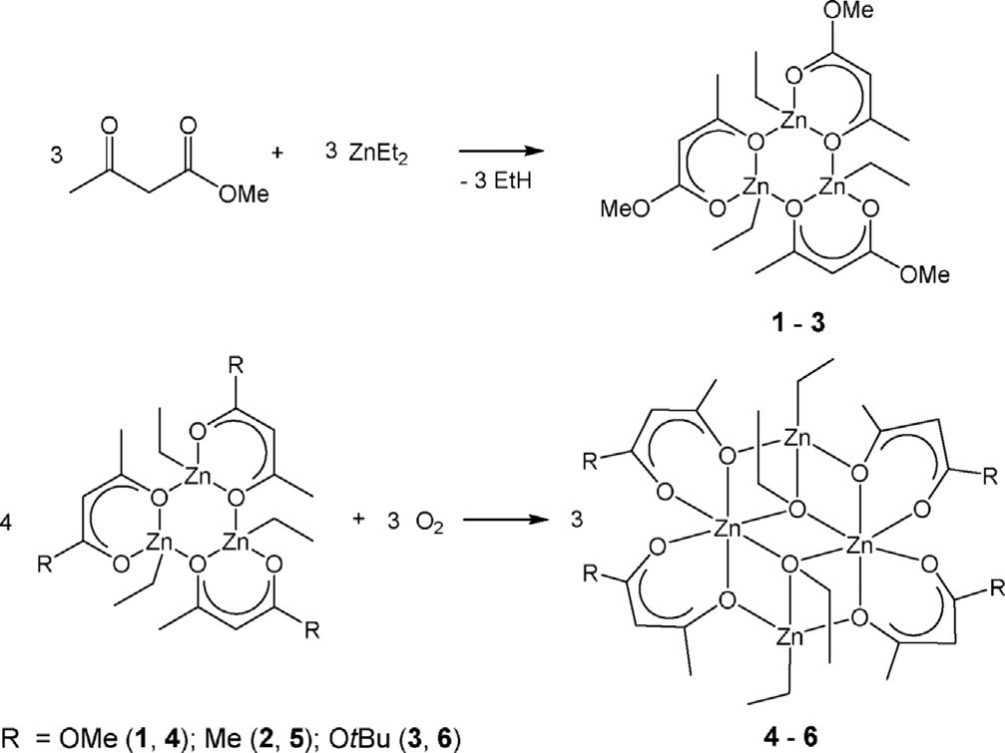
Synthesis of organozinc cyclic trimers **1**–**3** and selectively oxygenated zinc cluster complexes **4**–**6**.

Compound **1** crystallizes in the monoclinic space group *P*2_1_/*n* with three zinc centers, all exhibiting a distorted tetrahedral coordination (Figure 2). Each four‐coordinate zinc atom is linked to two *μ*_2_‐oxygen atoms and one terminal oxygen atom from the ligand and an ethyl group. The Zn−O bond distances in the Zn_3_O_3_ ring alternate between two types, the shorter Zn−O distance from the ligand [Zn(1)–O(O(1):2.0489(9) Å, Zn(2)–O(4):2.0247(9) Å, Zn(3)–O(7):2.0367(9) Å] and the longer bridging Zn−O lengths [Zn(1)–O(4):2.0710(10) Å, Zn(2)–O(7):2.0980(9) Å, Zn(3)–O(1):2.0698(10) Å], which is comparable to similar structures found in the literature.^17^ The distorted nature of the tetrahedral coordination around the Zn centers in **1** results largely from the constraints of the central Zn_3_O_3_ ring and the three outer ZnO_2_C_3_ rings formed from coordination to the bidentate ligands.

**Figure 2:**
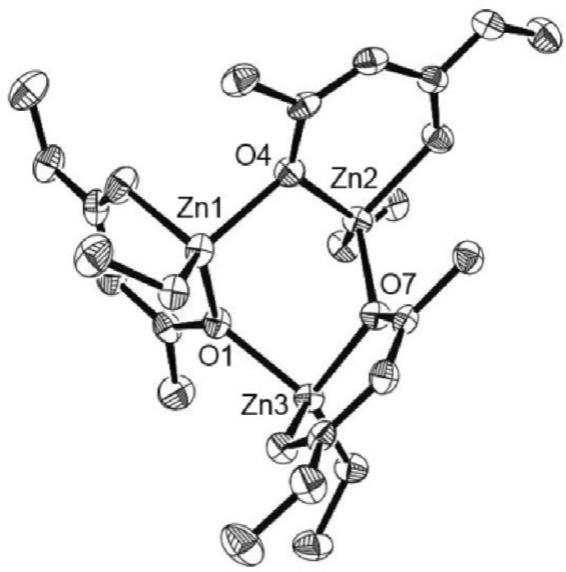
The molecular structure of **1**. H atoms omitted for clarity, thermal ellipsoids drawn at 50 % probability. Selected bond lengths [Å]: Zn(1)–O(1):2.0489(9), Zn(1)–O(4):2.0710(10), Z(1)–C(6) (ethyl): 1.9676(14).

For comparison with the previously reported ‘dimeric aggregate’ [{*t*BuZnO*t*Bu}_3_] (Figure 1 c), the closest distance between opposite zinc and oxygen atoms in the ring in **1** is Zn(1)–O(7), 3.042 Å, which is considerably larger than the comparable distance in [{*t*BuZnO*t*Bu}_3_] (2.164 Å),^16^ providing evidence of the cyclic trimeric nature of **1**.

The above data indicate that the use of a rigid bidentate *β*‐diketonate ligand facilitates the isolation of a novel type of cyclic trimeric organozinc complex (**1**) with a coordination environment of ZnCO_3_, as confirmed by crystallographic characterization. The monodentate *t*Bu groups used by Lewiński resulted in a metastable roof‐like trimer (or aggregated dimer), whereas the flexible amino alcoholates utilized by Molloy and co‐workers yielded oils, not solids, although NMR studies did suggest a cyclic trimeric center.^10^, ^15^, ^16^

The rationale behind this ligand choice firstly lies in the successful isolation of a novel structural type, but also in increasing the oxygen loading of the compounds (as zinc precursors can lead to oxygen‐deficient thin films).^19^ Increasing the oxygen content whilst maintaining volatility and suitably high reactivity such that decomposition can occur when required at lower temperature is a challenge in materials chemistry. As such, the following reactions involving the selective oxidation of compounds **1**–**3** were explored.

[{Zn(OC(R)CHC(Me)O)_2_Zn(Et)OEt}_2_] (R=OMe (**4**), Me (**5**), O*t*Bu (**6**)) were synthesized through the controlled addition of O_2_ to solutions of **1**–**3** (Scheme 1), presumably leading to selective insertion of dioxygen into one of the Zn−C bonds of the ethyl group, which, in turn, decomposes to a *μ*_2_‐OEt group in accordance with previous reports.^17^, ^18^ Compounds **4**–**6** were isolated in high yields (>80 %) and characterized by using spectroscopic techniques. Compounds **5** and **6** both crystallized out of concentrated solutions held at −18 °C as centrosymmetric clusters in the triclinic *P̄*1 space group with four zinc centers; two of which are unique and two are symmetrically generated about an inversion center (Figure 3). Compound **4** did not form crystals of suitable crystallographic quality; however, spectroscopic analysis confirms **4** is isostructural to **5** and **6** (see the Supporting Information).

**Figure 3:**
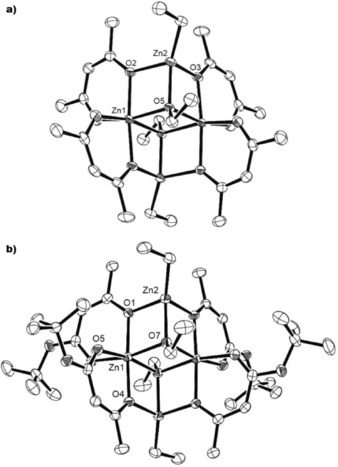
a) The molecular structure of **5**. H atoms omitted for clarity, thermal ellipsoids drawn at 50 % probability. Selected bond lengths [Å]: Zn(1)–O(1):2.0536(18), Zn(1)–O(5): 2.1217(16), Zn(2)–O(2): 2.0720(17), Zn(2)–O(5):2.0006(15). b) The molecular structure of **6**. H atoms omitted for clarity. Selected bond lengths [Å]: Zn(1)–O(1):2.0821(14), Zn(1)–O(7):2.1293(14), Zn(2)–O(1):2.0642(14), Zn(2)–O(7):2.0253(14). (Symmetry operator for both **5** and **6**: *i*=‐*X*, ‐*Y*, ‐*Z*).

The zinc centers in both **5** and **6** also have two different coordination modes: Zn(1) has a distorted octahedral geometry, whereas Zn(2) has a distorted tetrahedral geometry, similar to **1**. Deviations between bond lengths and angles in **5** and **6** are largely attributed to the increased bulk of the *t*Bu group in **6** compared to the Me group in **5**, and are in line with what would be expected.

In **5** and **6**, it can be observed that the octahedral coordination about zinc is distorted; this is likely caused by the steric constraints of the ZnO_2_C_3_ and Zn_2_O_2_ rings, a consequence of the bidentate ligands, just as with the tetrahedral distortion seen here in **1**.

Precursors of this type are of particular interest for technological applications, and as such we have explored the use of **5** as a single‐source precursor for the deposition of thin films of zinc oxide through aerosol‐assisted chemical vapor deposition (AACVD), details of which are included in the Supporting Information. Compound **5** was successfully employed as an AACVD precursor, resulting in the deposition of zinc oxide thin films by using an optimum substrate deposition temperature of 450 °C and annealing temperature of 600 °C.

In X‐ray photoelectron spectroscopy (XPS) of the film, the Zn 2p_1/2_ and 2p_3/2_ states were fitted by using a Gaussian/Lorentzian product distribution and appear, as expected, at binding energies of 1044.8 and 1021.7 eV, respectively, with an intensity ratio of 1:2 and an energy gap of 23.1 eV^20^ (Figure 4 a, inset). Depth profiling (Figure 4 a) revealed a zinc oxide thin film with low carbon contamination (<1 at % C) in the bulk of the film.

**Figure 4:**
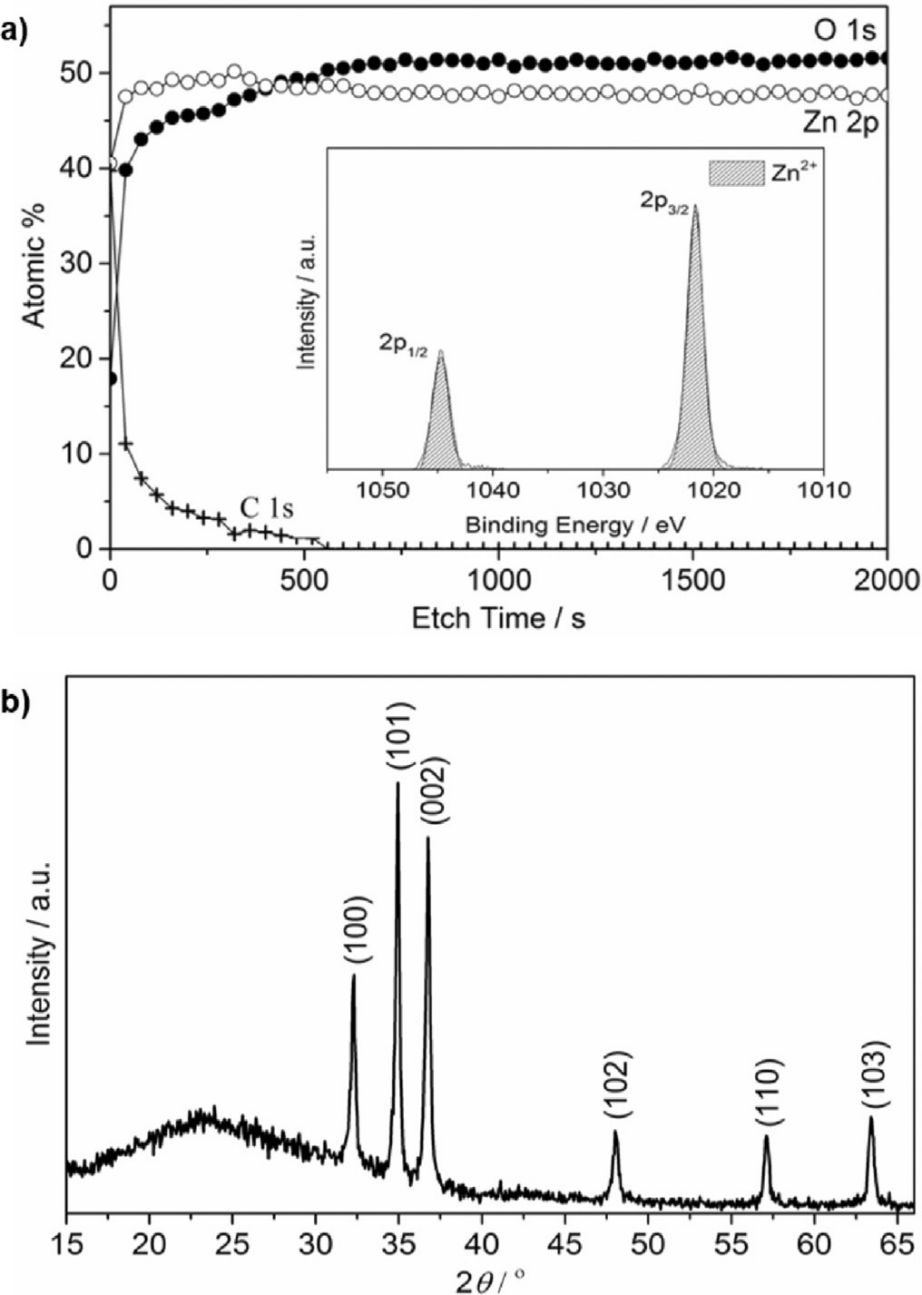
a) XPS depth profile, inset: the Zn 2p_1/2_ and 2p_3/2_ state peaks at 1044.8 and 1021.7 eV binding energy, respectively. b) XRD pattern for hexagonal wurtzite zinc oxide films.

Hexagonal wurtzite zinc oxide formation was further confirmed by using XRD (Figure 4 b). Scanning electron microscopy (SEM) revealed porous films formed of agglomerated rounded particles with diameters varying from 50 to 200 nm and a thickness of around 180 nm (Figure 5). The films were transparent (84 % in the visible‐light region) (Figure 6) and had an estimated band gap of 3.29 eV, as determined using the Tauc relation: *αhν=A*(*hν−E*_g_)^*n*^, where *α* is the molar extinction coefficient, *hν* is the energy of a particle of light, *A* is a constant, *E*_g_ is the band gap and *n* is 0.5 for a direct band gap (Figure 6, inset), consistent with literature values.^21^

**Figure 5:**
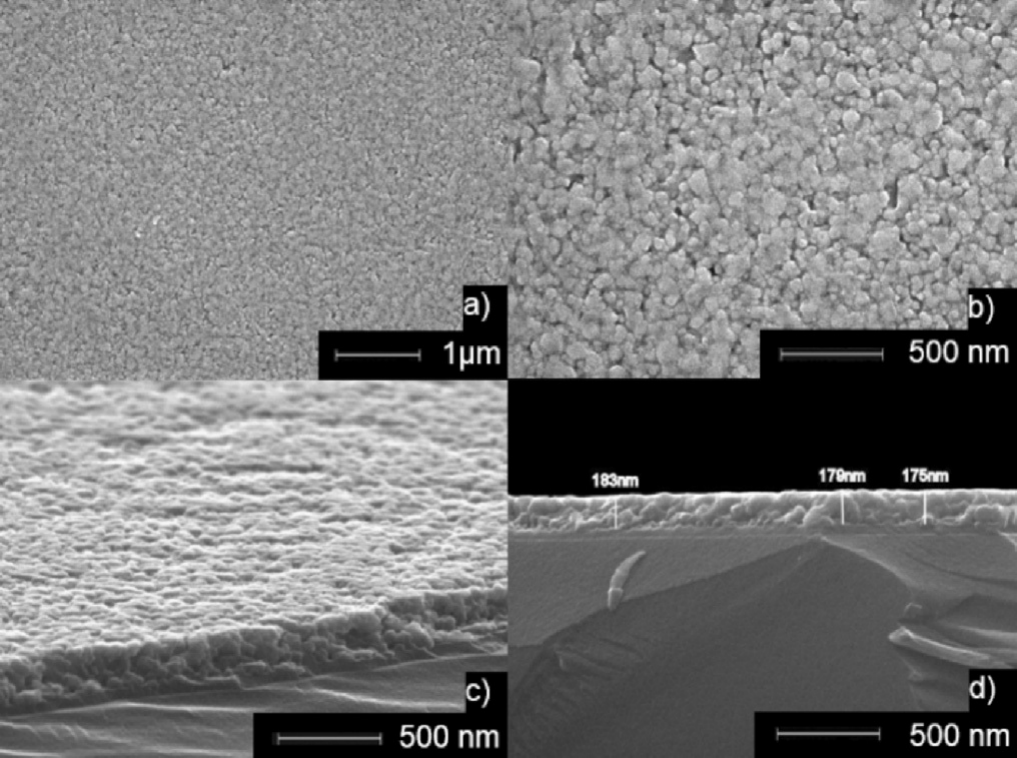
SEM images: a, b) plane view at ×20,000 and ×50,000 respectively; c, d) cross section at ×50,000 with an 80° and 90° tilt, respectively.

**Figure 6:**
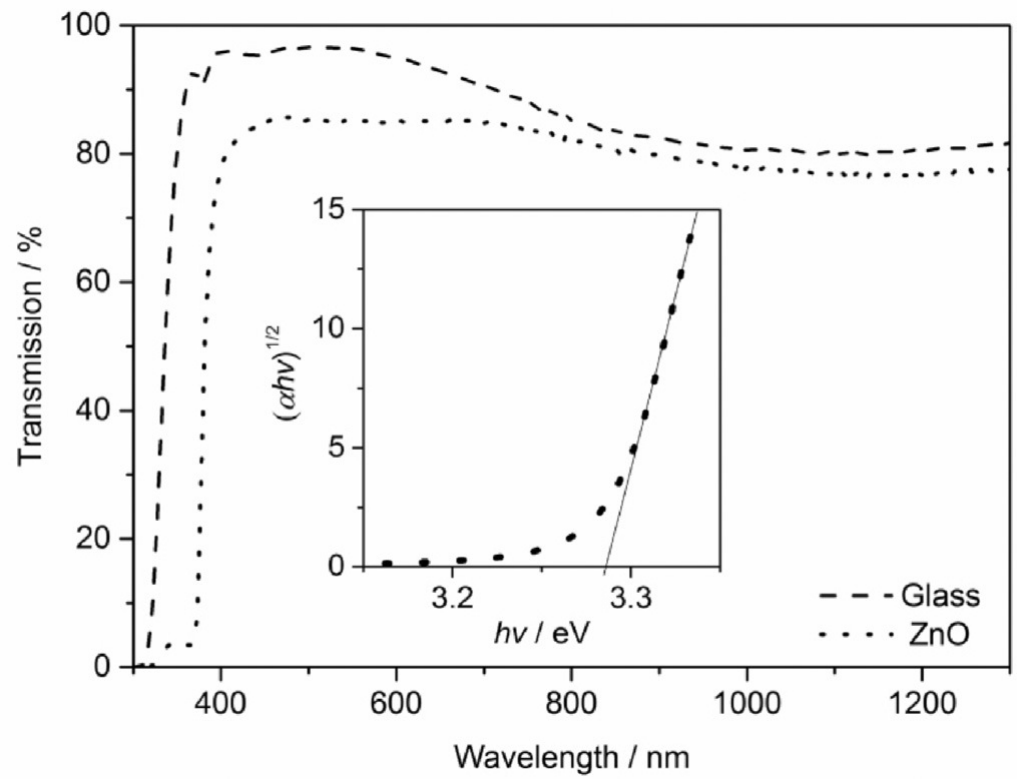
Transmission spectra of the deposited zinc oxide thin film. Inset: Tauc plot for band‐gap estimation.

In summary, we have demonstrated the solution‐based synthesis of the first structurally characterized cyclic trimer with a Zn_3_O_3_ core and ZnCO_3_ coordination environment (**1**), and spectroscopically characterized organozinc *β*‐diketonates of the type [{Zn(Et)(*β*‐diketonate)}_*n*_] (**2** and **3**). Through selective oxidation, we have subsequently shown the synthesis of [{Zn(OC(R)CHC(Me)O)_2_Zn(Et)OEt}_2_] (**4**–**6**). X‐ray crystallography of **5** and **6** revealed these complexes to have a face‐shared, corner‐removed, inversion‐related, bis‐heterocubane central motif. Of additional importance is that these materials can be used as precursors to thin films of zinc oxide; as exemplified in the successful deposition of hexagonal wurtzite zinc oxide thin films. We believe these results provide a new route, through careful ligand selection, to cyclic organozinc trimers, whose rarity was commented on by Power and co‐workers back in 1991.^22^ Further systematic studies will continue to expand the current collection of organozinc compounds of this type, and a more exhaustive study to optimize the film growth and physical properties of the zinc oxide will be undertaken to provide a platform for the construction of electronic devices.

## Experimental Section

Crystallographic/refinement data for compounds **1**, **5**, and **6** can be found in the Supporting Information.^23^

All manipulations were performed under a dry dinitrogen atmosphere by using standard Schlenk techniques. Hexane was stored in an alumina column and dried with anhydrous engineering equipment. Diethylzinc solution (1 m in hexanes) was obtained from Sigma Aldrich and used as supplied. Acetylacetone, methyl acetoacetate, and *tert*‐butyl acetoacetate were obtained from Sigma Aldrich, degassed, and stored over activated molecular sieves.

^1^H and ^13^C NMR spectra were obtained on a Bruker Avance III 600 cryo spectrometer and were recorded in C_6_D_6_. ^1^H and ^13^C chemical shifts are reported relative to SiMe_4_ (*δ* 0.00). Mass spectroscopy was performed on a Thermo Finnigan MAT900 XP operating in electron impact and chemical ionization modes. Single‐crystal X‐ray diffraction datasets were collected on a SuperNova (dual source) Atlas diffractometer by using either monochromated Cu K_α_ radiation (*λ*=1.54184 Å) or monochromated Mo K_α_ (*λ*=0.71073 Å).

### [{Zn(Et)(OC(OMe)CHC(Me)O)}_3_] (1)

Diethylzinc (9.76 mL, 1 m solution in hexanes, 9.76 mmol) was added to dry hexane (5 mL) at −78 °C. Dry methyl acetoacetate (1.13 g, 9.76 mmol) was added dropwise to the solution and stirred at RT for 48 h. Hexane was fully removed in vacuo, resulting in an off‐white solid. Off‐white crystals grew from a concentrated solution held at −18 °C. Yield: 1.86 g, 91 %; ^1^H NMR (600 MHz) *δ* (C_6_D_6_): 0.55 (q, 6 H, C*H*_2_CH_3_, *J*=8.0 Hz), 1.47 (t, 9 H, CH_2_C*H*_3_, *J*=8.00 Hz), 1.90 (s, 9 H, CC*H*_3_), 3.27 (s, 9 H, OC*H*_3_), and 4.95 ppm (s, 3 H, CC*H*C); ^13^C{^1^H} NMR (600 MHz) *δ* (C_6_D_6_): 1.0 (*C*H_2_CH_3_), 12.5 (CH_2_ *C*H_3_), 27.5 (C*C*H_3_), 51.1 (O*C*H_3_), 89.3 (C*C*HC), 174.0 (*C*OCH_3_), and 184.4 ppm (*C*CH_3_); anal. calcd. for C_21_H_36_O_9_Zn_3_: C 40.12, H 5.77; found: C 40.21, H 5.89.

### [{Zn(Et)(OC(Me)CHC(Me)O)}_3_] (2)

Diethylzinc (9.76 mL, 1 m solution in hexanes, 9.76 mmol) was added to dry hexane (5 mL) at −78 °C. Dry acetylacetone (0.98 g, 9.76 mmol) was added dropwise to the solution and stirred at RT for 48 h. Hexane was fully removed in vacuo, resulting in an off‐white solid. Yield: 1.71 g, 90 %; ^1^H NMR (600 MHz) *δ* (C_6_D_6_): 0.57 (q, 6 H, C*H*_2_CH_3_, *J*=8.2 Hz), 1.48 (t, 9 H, CH_2_C*H*_3_, *J*=8.2 Hz), 1.79 (s, 18 H, CC*H*_3_), and 5.03 ppm (s, 3 H, CC*H*C); ^13^C{^1^H} NMR (600 MHz) *δ* (C_6_D_6_): 1.0 (*C*H_2_CH_3_), 12.4 (CH_2_ *C*H_3_), 28.3 (C*C*H_3_), 102.0 (C*C*HC), and 193.1 ppm (*C*CH_3_); anal. calcd. for C_21_H_36_O_6_Zn_3_: C 43.44, H 6.25; found: C 43.09, H 6.77.

### [{Zn(Et)(OC(O*t*Bu)CHC(Me)O)}_3_] (3)

Diethylzinc (9.76 mL, 1 m solution in hexanes, 9.76 mmol) was added to dry hexane (5 mL) at −78 °C. Dry *tert*‐butyl acetoacetate (1.54 g, 9.76 mmol) was added dropwise to the solution and stirred at RT for 48 h. Hexane was fully removed in vacuo, resulting in an off‐white solid. Yield: 2.25 g, 91 %; ^1^H NMR (600 MHz) *δ* (C_6_D_6_): 0.37 (q, 6 H, C*H*_2_CH_3_, *J*=8.2 Hz), 1.31 (s, 27 H, C(C*H*_3_)_3_), 1.33 (t, 9 H, CH_2_C*H*_3_, *J*=8.2 Hz), 1.94 (s, 9 H, CC*H*_3_), and 4.90 ppm (s, 3 H, CC*H*C); ^13^C{^1^H} NMR *δ* (600 MHz) (C_6_D_6_): 3.4 (*C*H_2_CH_3_), 11.7 (CH_2_ *C*H_3_), 27.6 (C*C*H_3_), 28.4 (C(*C*H_3_)_3_), 80.9 *C*(CH_3_)_3_, 91.0 (C*C*HC), 173.8 (*C*OC(CH_3_)_3_), and 183.2 ppm (*C*CH_3_); anal. calcd. for C_30_H_54_O_9_Zn_3_: C 47.73, H 7.21; found: C 46.93; H 6.97.

### [{Zn(OC(OMe)CHC(Me)O)_2_Zn(Et)OEt}_2_] (4)

O_2_ (5 mL) was added to a solution of **1** and stirred for 15 min at −78 °C. The flask was purged with N_2_ and stirred at room temperature for 24 h. Off‐white crystals formed from a concentrated solution held at −18 °C. Yield: 1.85 g, 87 %; ^1^H NMR (600 MHz) *δ* (C_6_D_6_): 0.62 (q, 4 H, *J*=8.0 Hz, ZnC*H*_2_CH_3_), 1.33 (t, 6 H, *J*=7.0 Hz, OCH_2_C*H*_3_), 1.60 (t, 6 H, *J*=8.0 Hz, ZnCH_2_C*H*_3_), 1.90 (s, 12 H, CC*H*_3_), 3.33 (s, 12 H, OC*H*_3_), 3.87 (q, 4 H, *J*=7.0 Hz, C*H*_2_CH_3_), and 4.99 ppm (s, 4 H, CC*H*C); ^13^C{^1^H} NMR (600 MHz) *δ* (C_6_D_6_): 2.2 (Zn*C*H_2_CH_3_), 12.6 (ZnCH_2_ *C*H_3_), 19.7 (OCH_2_ *C*H_3_), 27.3 (C*C*H_3_), 50.6 (O*C*H_3_), 61.3 (O*C*H_2_CH_3_), 87.0 (C*C*HC), 173.6 (*C*OCH_3_), and 183.3 ppm (*C*CH_3_); anal. calcd. for C_28_H_48_O_14_Zn_4_: C 38.64, H 5.56; found: C 38.45, H 5.64. MS: *m*/*z* [M−Zn_2_O_5_C_13_H_27_]^+⋅^: 474.87; [M−Zn_3_O_8_C_18_H_34_]^+⋅^: 294.97.

### [{Zn(OC(Me)CHC(Me)O)_2_Zn(Et)OEt}_2_] (5)

O_2_ (5 mL) was added to a solution of **2** and stirred for 15 min at −78 °C. The flask was purged with N_2_ and stirred at room temperature for 24 h. Off‐white crystals formed from a concentrated solution held at −18 °C. Yield: 1.66 g, 84 %; ^1^H NMR (600 MHz) *δ* (C_6_D_6_): 0.69 (br, 4 H, ZnC*H*_2_CH_3_), 1.42 (t, 6 H, *J*=7.0 Hz, OCH_2_C*H*_3_), 1.61 (br, 6 H, ZnCH_2_C*H*_3_), 1.79 (s, 24 H, CC*H*_3_), 3.80 (q, 4 H, *J*=7.0 Hz, OC*H*_2_CH_3_), and 5.06 ppm (s, 4 H, CC*H*C); ^13^C{^1^H} NMR (600 MHz) *δ* (C_6_D_6_): 2.7 (Zn*C*H_2_CH_3_), 12.9 (ZnCH_2_ *C*H_3_), 20.1 (OCH_2_ *C*H_3_), 28.1 (C*C*H_3_), 61.1 (O*C*H_2_CH_3_), 100.6 (C*C*HC), and 193.1 ppm (*C*CH_3_); anal. calcd. for C_28_H_48_O_10_Zn_4_: C 41.71, H 6.00; found: C 42.06, H 5.92. MS: *m*/*z* [M+C_3_H_5_]^+⋅^: 847.04; [M−C_8_H_20_]^+⋅^: 691.11; [M−Zn_2_O_4_C_13_H_27_]^+⋅^: 426.91; [M−Zn_3_O_6_C_18_H_34_]^+⋅^: 263.00.

### [{Zn(OC(O*t*Bu)CHC(Me)O)_2_Zn(Et)OEt}_2_] (6)

O_2_ (5 mL) was added to a solution of **3** and stirred for 15 min at −78 °C. The flask was purged with N_2_ and stirred at room temperature for 24 h. Off‐white crystals formed from a concentrated solution held at −18 °C. Yield: 2.09 g, 83 %; ^1^H NMR (600 MHz) *δ* (C_6_D_6_): 0.58 (q (br), 4 H, ZnC*H*_2_CH_3_), 1.44 (s, 36 H, C(C*H*_3_)_3_), 1.51 (t (br), 6 H, OCH_2_C*H*_3_), 1.66 (t, 6 H, *J*=8.1 Hz, ZnCH_2_C*H*_3_), 1.92 (s, 12 H, CC*H*_3_), 4.02 (q, 4 H, *J*=7.1 Hz, OC*H*_2_CH_3_), and 4.96 ppm (s, 4 H, CC*H*C); ^13^C{^1^H} NMR (600 MHz) *δ* (C_6_D_6_): 1.8 (Zn*C*H_2_CH_3_), 13.1 (ZnCH_2_ *C*H_3_), 19.8 (OCH_2_ *C*H_3_), 27.4 (C*C*H_3_), 28.7 (C(*C*H_3_)_3_), 61.4 (O*C*H_2_CH_3_), 79.7 (*C*(CH_3_)_3_), 88.7 (C*C*HC), 173.4 (*C*OC(CH_3_)_3_), and 182.1 ppm (*C*CH_3_); anal. calcd. for C_40_H_72_O_14_Zn_4_: C 46.26, H 6.99; found: C 46.43, H 7.27. MS: *m*/*z* [M−Zn_2_O_5_C_16_H_33_]^+⋅^: 601.05; [M−Zn_3_O_8_C_24_H_46_]^+⋅^: 379.04.

### Thin‐Film Deposition and Analysis

Films were deposited onto float‐glass substrates with a 25 nm barrier layer of crystalline SiO_2_. [(Zn(OC(Me)CHC(Me)O)_2_Zn(Et)O(Et))_2_] (**5**) (0.7 g, 0.87 mmol) was dissolved in dry toluene (30 mL) under N_2_ and stirred for 10 min. Thin films were deposited by using optimal conditions of a N_2_ flow rate of 1.2 L min^−1^, a substrate temperature of 450 °C, and annealing in air for 5 h at 600 °C. XRD was performed using a Bruker D8 Discover X‐ray diffractometer by using monochromatic Cu K_α1_ and Cu K_α2_ radiation of wavelengths 1.54056 and 1.54439 Å, respectively, emitted in an intensity ratio of 2:1 with a voltage of 40 kV and a current of 40 mA. SEM was performed by using a Philips XL30 FEG operating in plan and cross‐section mode with an electron beam accelerating energy of 30 kV. XPS surface and depth profiling was performed by using a Thermo Scientific K‐Alpha XPS system with monochromatic Al K_α_ radiation at 1486.6 eV as the X‐ray source. Etching was achieved by using an Ar‐ion etch beam at 1 KeV with a current of 1.51 μA. CasaXPS software was used to analyze the data with binding energies referenced to an adventitious C 1s peak at 284.8 eV. UV/Vis/NIR transmission spectra were recorded by using a PerkinElmer Lambda 950 spectrometer in the range of 300–1400 nm with an air background.

## Supporting information

As a service to our authors and readers, this journal provides supporting information supplied by the authors. Such materials are peer reviewed and may be re‐organized for online delivery, but are not copy‐edited or typeset. Technical support issues arising from supporting information (other than missing files) should be addressed to the authors.

Supplementary
