# Supplementary material for: Synthesis of Trimeric Organozinc Compounds and their Subsequent Reaction with Oxygen
Source: ChemistryOpen. 2016 Jul 15;5(4):301–5. doi: 10.1002/open.201600040 (PMC4981048; doi:10.1002/open.201600040)
Supplement: Supplementary file 1 — Supplementary [file OPEN-5-301-s001.pdf]

ChemistryOPEN

Supporting Information

© 2016 The Authors. Published by Wiley-VCH Verlag GmbH & Co. KGaA, Weinheim

Synthesis of Trimeric Organozinc Compounds and their Subsequent Reaction with Oxygen

Joe A. Manzi, Caroline E. Knapp, Ivan P. Parkin, and Claire J. Carmalt*[a]

open_201600040_sm_miscellaneous_information.pdf

S1. Crystallographic Data

Olex2[1] was used to solve the three structures. For **1**, the SHELXS[2] structural solution programme using Direct Methods was employed and the structure refined with SHELXL[2] using Least Squares minimisation. For **5** and **6**, the Superflip[3–5] structural solution programme using Charge Flipping was employed and the structure refined with olex2.refine[6] using Gauss-Newton minimisation.

Table 1. Crystallographic and structure refinement data for compounds 1, 5 and 6.

Zinc Complex	**1**	**5**	**6**
λ/Å	1.54184	0.71073	0.71073
Temperature/K	150.05(10)	150.05(10)	150.08(13)
Crystal System	Monoclinic	Triclinic	Triclinic
Space Group	$P2_1/n$	$P\bar{1}$	$P\bar{1}$
Volume/Å^3	2647.74(3)	838.34(10)	2455.44(12)
a/Å	14.33392(8)	9.3721(7)	11.2864(2)
b/Å	9.09638(5)	9.9549(6)	13.2409(4)
c/Å	20.47334(14)	10.5962(7)	17.7760(5)
α/°	90	99.361(5)°	110.060(3)
β/°	97.3126(6)	113.292(6)°	90.455(2)
γ/°	90	104.798(6)°	99.460(2)
Z	4	2	2
ρ_{calc}g/cm^3	1.577	1.597	1.405
μ/mm^{-1}	3.553	2.877	1.987
F(000)	1296.0	416.0	1088.0
2*θ* Data Collection Range/°	7.122 – 147.316	6.314 – 58.304	5.936 – 58.722
Index Ranges	$-17 \le h \le 17$, $-11 \le k \le 11$, $-23 \le l \le 24$	$-11 \le h \le 12$, $-12 \le k \le 12$, $-14 \le l \le 14$	$-15 \le h \le 15$, $-17 \le k \le 17$, $-24 \le l \le 24$
Reflections Collected	41363	12392	35990
Independent Reflections	5292 R_{int} = 0.0276, R_σ = 0.0120	4072 R_{int} = 0.0344, R_σ = 0.0392	11908 R_{int} = 0.0415, R_σ = 0.0428
Data/Restraints/Parameters	5292 / 0 / 307	4072 / 0 / 196	11908 / 0 / 543
Goodness-of-fit on F^2	1.074	1.072	1.047
Final *R* Indexes ($I \ge 2\sigma(I)$)	R_1 = 0.0195, wR_2 = 0.0516	R_1 = 0.0328, wR_2 = 0.0707	R_1 = 0.0350, wR_2 = 0.0783
Final *R* Indexes (All Data)	R_1 = 0.0206, wR_2 = 0.0522	R_1 = 0.0433, wR_2 = 0.0771	R_1 = 0.0453, wR_2 = 0.0844
Largest Diff. Peak / Hole/eÅ^{-3}	0.23 / -0.27	0.50 / -0.41	1.09 / -0.64

[1] O. V. Dolomanov, L. J. Bourhis, R. J. Gildea, J. A. K. Howard, H. Puschmann, *J. Appl. Crystallogr.* **2009**, *42*, 339–341.
[2] G. M. Sheldrick, *Acta Crystallogr. A* **2008**, *64*, 112–122.
[3] L. Palatinus, G. Chapuis, *J. Appl. Crystallogr.* **2007**, *40*, 786–790.
[4] L. Palatinus, A. van der Lee, *J. Appl. Crystallogr.* **2008**, *41*, 975–984.
[5] L. Palatinus, S. J. Prathapa, S. van Smaalen, *J. Appl. Crystallogr.* **2012**, *45*, 575–580.
[6] L. J. Bourhis, O. V. Dolomanov, R. J. Gildea, J. A. K. Howard, H. Puschmann, *Acta Crystallogr. Sect. Found. Adv.* **2015**, *71*, 59–75.
